# Smartphones for community health in rural Cambodia: A feasibility study

**DOI:** 10.12688/wellcomeopenres.13751.1

**Published:** 2018-06-12

**Authors:** Pengby Ngor, Lisa J. White, Jeremy Chalk, Yoel Lubell, Cecelia Favede, Phaik-Yeong Cheah, Chea Nguon, Po Ly, Richard J. Maude, Siv Sovannaroth, Nicholas P. Day, Susanna Dunachie

**Affiliations:** 1 Cambodian National Malaria Center, National Centre for Parasitology, Entomology and Malaria Control, Phnom Penh, Cambodia; 2 Mahidol-Oxford Tropical Medicine Research Unit, Faculty of Tropical Medicine, Mahidol University, Bangkok, Thailand; 3 Centre for Tropical Medicine, Nuffield Department of Medicine, University of Oxford, Oxford, UK; 4 Harvard TH Chan School of Public Health, Harvard University, Boston, USA; 5 Peter Medawar Building for Pathogen Research, University of Oxford, Oxford, UK

**Keywords:** malaria, smartphone, technology, m-health, community

## Abstract

**Background: **Village Malaria Workers (VMWs) are lay people trained to provide a valuable role in frontline testing and treatment of malaria in rural villages in Cambodia. Emergence of artemisinin-resistant malaria highlights the essential role of such VMWs in surveillance and early treatment of malaria. Smartphone technology offers huge potential to support VMWs in isolated and resource-poor settings.

**Methods: **We investigated the feasibility of issuing established VMWs with a smartphone, bespoke Android application and solar charger to support their role. 27 VMWs in Kampong Cham and Kratie provinces participated.

**Results: **26/27 of the smartphones deployed were working well at study completion twelve months later. Interviews with VMWs using quantitative and qualitative methods revealed pride, ease of use and reports of faster communication with the smartphone. VMWs also expressed a strong wish to help people presenting with non-malarial fever, for which further potential supportive smartphone applications are increasingly available.

**Conclusions: **As a result of this pilot study, two smartphone based reporting systems for malaria have been developed at the Cambodian National Malaria Center, and the programme is now being extended nationwide. The full code for the smartphone application is made available to other researchers and healthcare providers with this article. Smartphones represent a feasible platform for developing the VMW role to include other health conditions, thus maintaining the relevance of these important community health workers.

## Introduction

More than 3 billion people, including 70% of the world’s poorest people, live in rural areas ^1^, where access to skilled medical care may be difficult ^2^, especially during the rainy season. Trained lay people in target communities can offer, via supported schemes, a valuable contribution to diagnosis and management of medical conditions such as malaria, childbirth and childhood diarrhoea ^3^. However, giving sufficient support to such community workers, including training updates, diagnostic support, stock control and data collection is challenging. Mobile phone technology provides the potential to improve the range and quality of services delivered by community health workers ^4^, but the majority of published literature on feasibility in developing countries is focussed on standard mobile phones (“feature phones”) rather than the next generation of smartphones.

The Cambodian National Malaria Center (CNM) has an established network of Village Malaria Workers (VMWs) across 19 provinces covering a population of approximately 1.5 million people ^5^. VMWs review villagers with fever, test them for malaria with a rapid test (SD Bioline Malaria Ag Pf/Pan; *Standard Diagnostics Inc*, Gyeonggi-do, Republic of Korea) and treat those with positive results with artemesinin-based antimalarial therapy. Rapid case reporting by coded text message (short message service; SMS) from feature phones has been established by CNM. We set out to evaluate the feasibility of replacing the feature phone part of this reporting system with smartphones and a bespoke Android-powered app (smartphone application). 

## Methods

**Figure 1.**
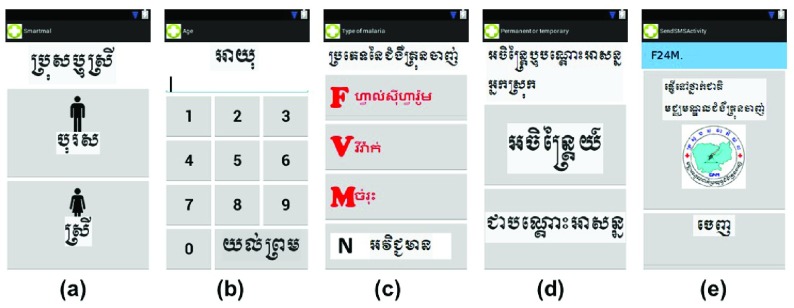
Screenshots of bespoke app for data collection allowing data entry. Screenshots show gender ( **a**), age ( **b**), malaria rapid test result ( **c**), residency status (permanent villager or mobile migrant) ( **d**), and exit screen to send data including GPS signal ( **e**).

**Figure 2.**
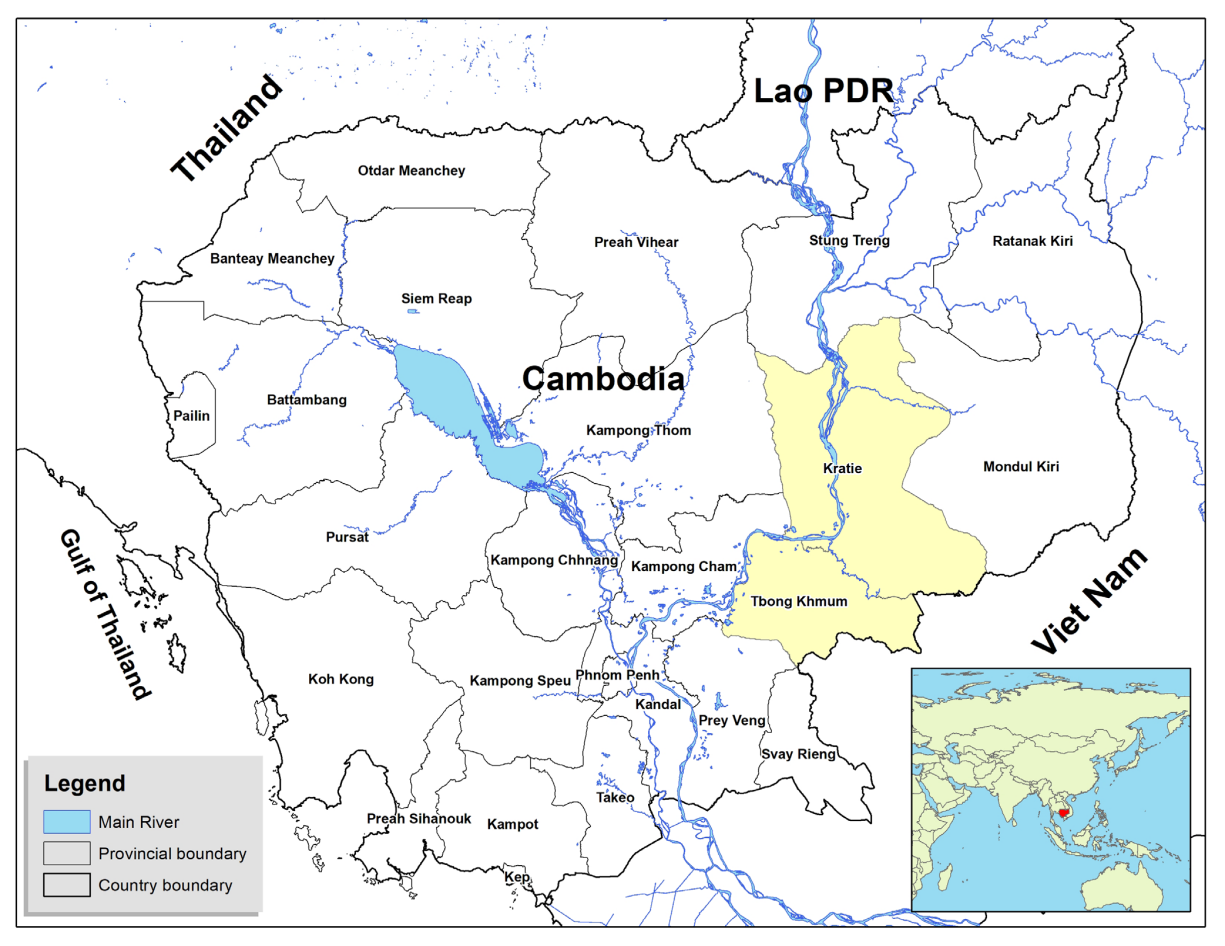
Map of Cambodia showing location of research. Kampong Cham and Kratie provinces are shaded yellow (source: Cambodia National Malaria Center, adapted from http://www.un.org/Depts/Cartographic/map/profile/cambodia.pdf. The boundaries and names shown and the designations used on this map do not imply the expression of any opinion whatsoever on the part of the Ministry of Health of Cambodia concerning the legal status of any country, territory, city, or area or of its authorities, or concerning the delimitation of its frontiers or boundaries).

A new smartphone application (“app”) was designed and built for Android phones using the Open Data Kit platform version 1.3 ^6, 7^. All instructions for installation, configuration, implementation and operation are explained in detail with examples on the Open Data Kit website. The minimum system requirements for the smartphone are Android devices 4.0 and above, and the phone should be connected to the Google App Engine hosting platform. Code is available ^18^. The app was designed to be simple to use, not require current network access to be operational, and allowed the VMW to enter case report data in an intuitive way using Khmer language ( Figure 1). The information recorded by the app and sent to CNM was as shown in the screenshots in Figure 1, namely gender, age, malaria rapid test result, and residency status (permanent villager or mobile migrant). Experienced VMWs in Kampong Cham and Kratie provinces ( Figure 2) were trained to use the new smartphone system. The numbers were limited by the cost of the phones. This was a service improvement project for the VMW network and ethical permission was not sought by the authors.

VMWs were called to a meeting and the proposed study explained by CMN staff. All VMWs gave verbal consent to participate in the study, and then VMWs received one smartphone (Acer Liquid Z3, *Acer*, Virginia, USA) and one solar power charger (model LG-FD12D10, *TBS Solar Solution Center*, Phnom Penh, Cambodia) each (total cost $175 per VMW), alongside a 3-hour training session delivered at the local Health Centre by CNM staff. A coded SMS was generated automatically and sent to CNM using 2G or 3G telecommunications networks, which have an estimated 99% coverage in Cambodia ^8^, including all the villages under study. The project ran for twelve months from September 2014.

Prior experience of smartphones for each VMW and their experiences in the role of VMW was established by in-person questionnaire at the outset of the study using closed questions (Pre-implementation questionnaire, Supplementary File 1). Survival of the smartphone at 12 months was determined by inspection by CNM staff, and quantitative and qualitative data on VMW’s experiences were collected by in-person questionnaire conducted by CNM health workers using open questions, translated from Khmer to English language and analysed for key emerging themes ( Supplementary File 2). The information captured by the smartphone app and sent to CMN was available to the researchers. Outcome measures were 1) Survival of smartphones and chargers over twelve months and 2) Acceptability of smartphone use by VMWs using verbal questionnaires administered in-person by CMN staff.

## Results

**Figure 3.**
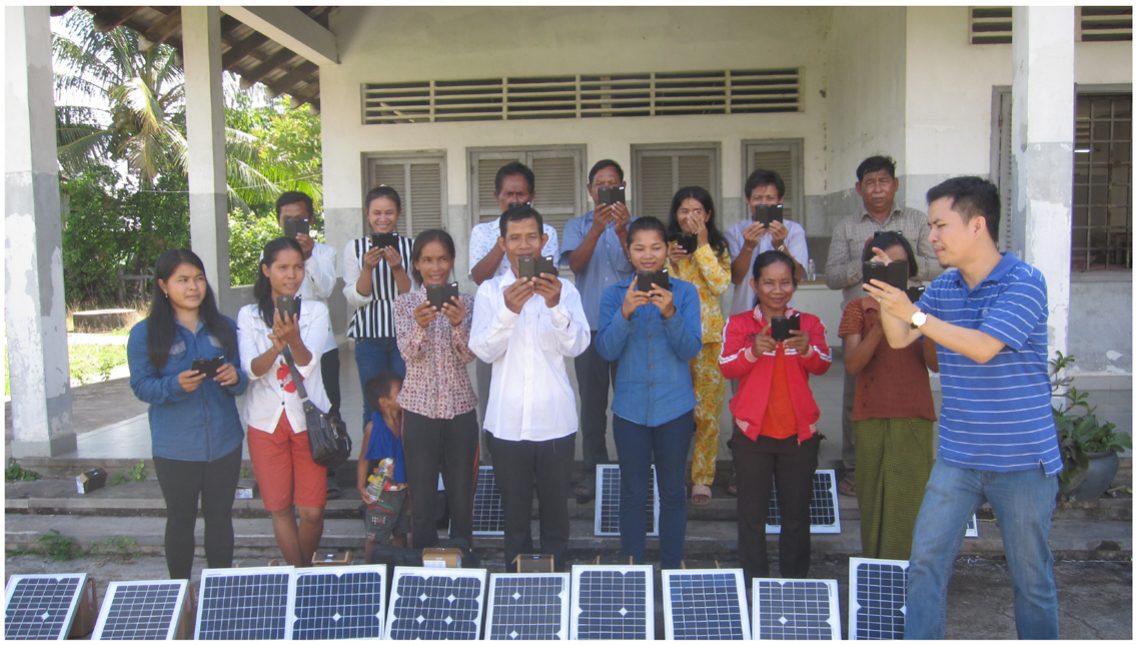
Village Malaria Workers (VMW) receive training. A group of VMWs in Kratie Province receiving training in the use of smartphones for the project at Sambo Health. All individuals gave their consent for the publication of this image.

Twenty-seven experienced VMWs in Kampong Cham and Kratie provinces were trained to use the new smartphone system ( Figure 3). VMWs in Kampong Cham province had previous experience with feature phones, while those in Kratie province had no experience of using phones in their work. Populations were broadly similar in the two provinces in demographics and socio-economic factors. The average distance of each village to the nearest health centre was 7.53km (range 2.9–10.3 km) for Kampong Cham province, and 16.9km (range 4.57–39.1 km) for Kratie province. Road access to the health centre during the rainy season was typically considered impossible or extremely difficult.

The results of the pre-implementation in-person questionnaire at the outset of the study are shown in Table 1 and Dataset 2. 15/27 (56%) of the VMWs were female and the mean age was 39 years (range 20 – 62). 100% were literate (a requirement for being a VMW) with the highest level of education completed being primary school level only for 81%, and all had been in post for more than three years. 85% already owned a feature phone, but only three VMWs (11%) owned a smartphone and the majority (85%) had never used one. The baseline survey of expectations showed 78% were expecting the smartphones to make their work easier and 70% were excited/proud to be using them. Motivations for being a VMW were to help others (78%), stop malaria (63%), attain new skills (37%), gain respect from their community (33%), financial/other incentives, with payment of $20 USD per month (33%), and to gain access to free health centre services (26%). Problems faced by VMWs included poor adherence or cooperation from patients (33%), not enough time (27%), long distance to the patient’s home (22%), lack of transportation (22%), not enough support from the Health Centre (22%), lack of incentives (19%), too many other tasks (15%), and misunderstanding about malaria in the village (15%).

**Table 1.** Baseline demographics and views of Village Malaria Workers in the study.

	Kampong Cham	Kratie	Total
**Demographics**			
Total, n	14	13	**27**
No. female, n (%)	7 (50)	8 (62)	**15 (56)**
Mean age in years (range)	38 (20 – 60)	40 (21 – 62)	**39 (20 – 62)**
Education beyond primary, n (%)	1 (7)	4 (31)	**5 (19)**
**Prior phone use**			
Already own feature phone, n (%)	13 (93)	10 (77)	**23 (85)**
Have used feature phone before, n (%)	14 (100)	10 (77)	**24 (88)**
Already own smartphone, n (%)	3 (21)	0 (0)	**3 (11)**
Have used smartphone before, n (%)	4 (29)	0 (0)	**4 (15)**
**Expectations**			
Thinks it will make VMW role easier, n (%)	14 (100)	7 (54)	**21 (78)**
Excited, n (%)	8 (57)	1 (8)	**9 (33)**
Proud, n (%)	5 (36)	5 (38)	**10 (37)**
Worried about losing it, n (%)	4 (29)	3 (23)	**7 (26)**
Worried about difficulties charging it, n (%)	0 (0)	1 (8)	**1 (4)**
Worried about difficulties operating it, n (%)	0 (0)	1 (8)	**1 (4)**
**Motivation for being a VMW**			
To help others, n (%)	11 (79)	10 (77)	**21 (78)**
To contribute to malaria control, n (%)	13 (93)	4 (31)	**17 (63)**
To gain respect within their community, n (%)	9 (64)	0 (0)	**9 (33)**
To access free health care, n (%)	7 (50)	0 (0)	**7 (26)**
Financial / other incentives, n (%)	7 (50)	2 (15)	**9 (33)**
To attain new skills, n (%)	8 (57)	2 (15)	**10 (37)**
**Challenges faced as a VMW**			
Long distance to travel to a patient’s home, n (%)	1 (7)	5 (38)	**6 (22)**
Lack of transport, n (%)	2 (14)	4 (31)	**6 (22)**
Not enough time, n (%)	6 (43)	1 (8)	**7 (26)**
Lack of incentives, n (%)	4 (29)	1 (8)	**5 (19)**
Poor patient adherence / co-operation, n (%)	8 (57)	1 (8)	**9 (33)**
Misunderstanding about malaria, n (%)	3 (21)	1 (8)	**4 (15)**
Too many other tasks	3 (21)	1 (8)	**4 (15)**
Not enough support from the Health Centre, n (%)	5 (36)	1 (8)	**6 (22)**

At study completion 12 months later, a follow-up survey (Dataset 3) showed 26/27 smartphones (96%) and 27/27 solar chargers (100%) issued to VMWs were working well. One USB connector on the smartphone was broken so it could not be charged. In addition, one smartphone was stolen during the training session prior to issue. CNM reported that training was quicker and easier to conduct than for paper or feature phone data collection methods. 20/27 (74%) VMWs reported cases using smartphones - 14/14 (100%) VMWs from Kratie province (no previous feature phone experience) and 6/13 (46%) from Kampong Cham province (with previous feature phone experience) reported data. For the 7 VMWs who did not report cases using smartphone, 2 recorded malaria cases by paper records, while for the remaining 5 there were no malaria cases reported. Negative test results were not routinely reported by SMS in accordance to CNM policy (due to funding limits on the number of SMSs allocated to the scheme).

All 27 VMWs were interviewed by CNM health workers at the end of the study using a semi-structured format with a topic guide, to evaluate experience and attitudes (Supplementary Data 3). Three main themes emerged: enjoyment and pride of owning the smartphone, finding it easier to use than a feature phone for their role (Kampong Cham province), and thinking that it allowed faster communication. VMWs were asked about what people in their village said about the smartphone and the key themes were curiosity, envy, surprise and being aware of the phones attracting a lot of attention in the village.

The pre-implementation questionnaire found evidence that in villages with much reduced malaria incidence, many villagers who have fever are no longer consulting VMWs. 59% of VMWs reported that only ‘some’ villagers would see them with a fever. In a 3-month period (Sep-Nov 2014) the information captured by the smartphone app showed tests on 89 febrile villagers were reported in the 13 villages in Kampong Cham province (mean 7 per village, range 3–10) including 19 malaria positive cases. In contrast, in the same period in Kratie, 688 tests on febrile villagers were reported (mean 49 per village, range 25–75) including 371 malaria positive cases. We also found evidence of frustration amongst VMWs that they were unable to help those with non-malarial fever, with 17/27 (63%) wanting to learn more about healthcare.

## Discussion

This pilot study demonstrates the feasibility of using a smartphone with a bespoke app to support community health workers in rural villages in low-income countries. VMWs reported pride and enjoyment at having use of a smartphone for their role, and felt it allowed faster communication. The smartphone raised the profile of the VMW in their village.

A previous systematic review of 42 studies of using mobile technology in developing countries ^9^ has shown the feasibility and some descriptive evidence of effectiveness of delivering healthcare using predominantly feature phones by professional front line workers such as midwives, pharmacists, nurses, doctors and some Community Health Workers (CHWs). Innovative studies are emerging demonstrating the role of smartphone apps delivered by professional healthcare workers in developing countries for health such as hearing screening in South Africa ^10^ and visual acuity testing in Kenya ^11^. In addition, use of a smartphone-based electronic decision support system by CHWs has shown promise for cardiovascular management in rural Tibet and India ^12^. The current study expands the literature to demonstrate feasibility and acceptability in an impoverished population of Cambodian CHWs, to support malaria control strategies.

The emergence and spread of artemisinin resistant malaria in Cambodia ^13– 15^ is a major threat to global health, thus control of malaria in rural Cambodia is of the utmost importance. We identified low numbers of malaria cases detected by the VMWs in this study, with many VMWs feeling frustrated at being unable to help those with non-malaria fever under the current scheme. After a number of ‘negative’ tests people might not consult the VMW with a fever anymore, thus the sentinel role of the VMW scheme in surveillance for malaria is reduced. This undermines malaria control programs because future outbreaks of malaria could go unnoticed and then spread to surrounding regions.

Based on the evidence of this study and the national capacity built in the relevant coding skills, the co-authors at the national control program in Cambodia are now developing two smartphone based reporting systems for malaria. At the VMW level, the application developed in this study has been extended to record all the case data recorded by VMW in paper records. This information is then uploaded directly to the national malaria database after entry. There is a plan to install this application on 3300 smartphones for introduction in 14 districts, 22 districts, 9 districts in 2018, 2019, 2020, respectively, with a view to paperless reporting in the future. An additional feature of an SMS alerting system has been included in selected pilot areas, which immediately reports every new case to every level of the surveillance system. This system has been integrated into a reactive case detection intervention in the piloted areas. At the health centre level, a similar application has been developed which focuses on case reports and stock-out alerts for treatments and diagnostics. This application has been installed on tablets in 816 health centres covering the entire malaria endemic area of Cambodia.

The limitations of this study are the small numbers of VMW involved, the short duration of follow up and the restricted amount of information collected on follow-up. These restrictions are due to the project being conducted with no extra resources available. In addition, the extension of the project by the CNM due to excellent informal feedback meant longer follow up was no longer feasible or deemed necessary.

The VMW scheme in Cambodia urgently needs to be enhanced and extended to be the front line in monitoring for drug resistance and future epidemics. To achieve this, and to maintain its relevance to local communities, it should also become a service for other health problems, transforming VMWs into CHWs. These CHWs would treat or refer patients with other health concerns making them the first point of contact in a village. We have demonstrated smartphone technology to be a robust platform for delivery of these services in the local language, enabling use of voice calls, SMS, photography, video, audio and GPS location tracking. As telecommunication network coverage and mobile use expand globally, exploitation of smartphone applications hold growing promise to tackle the world’s greatest health issues. This highly successful pilot project of introducing smartphone-based reporting for malaria has allowed the CNM to develop the platform for implementation. 

## Data and software availability

Dataset 1: Baseline Demographics and Views VMW survey available on Figshare, https://doi.org/10.6084/m9.figshare.6326723 ^16^

Dataset 2: Post Implementation Survey VMWs available on Figshare, https://doi.org/10.6084/m9.figshare.6327221 ^17^

Data are available under the terms of the Creative Commons Zero "No rights reserved" data waiver (CC0 1.0 Public domain dedication).

Archived source code of Smartphone App at time of publication: https://doi.org/10.6084/m9.figshare.6353210 ^18^

License: CC0

## Consent

All Village Malaria Workers consented to participate in the questionnaires. This study was part of a service improvement project involving Village Malaria Workers working for the Cambodia National Malaria Center, and specific written consent to participate in the study was not deemed necessary.

## Supplementary material

Supplementary File 1: Pre-implementation survey: Baseline Demographics and Views.

Supplementary File 2: Post-implementation survey: VMWs’ experience of using smartphones.

## References

[1] United Nations DoEaSA, Population Division: World Urbanization Prospects: The 2014 Revision. New York, USA;2015 Reference Source

[2] WHO: World Health Statistics 2014. Geneva;2014 Reference Source

[3] Perry H Zulliger R: How Effective are Community Health Workers?2012 Reference Source

[4] Braun R Catalani C Wimbush J: Community health workers and mobile technology: a systematic review of the literature. *PLoS One.* 2013;8(6):e65772. 10.1371/journal.pone.0065772 23776544 PMC3680423

[5] Cox J Sovannaroth S Dy Soley L: Novel approaches to risk stratification to support malaria elimination: an example from Cambodia. *Malar J.* 2014;13:371. 10.1186/1475-2875-13-371 25233886 PMC4177243

[6] Hartung C Lerer A Anokwa Y: Open data kit: tools to build information services for developing regions. *Proceedings of the 4th ACM/IEEE International Conference on Information and Communication Technologies and Development.*London, United Kingdom. 2369236: ACM;2010;1–12. Reference Source

[7] Open Data Kit. Accessed 10th August 2017. Reference Source

[8] TheGlobalEconomy.com: Cambodia: Mobile network coverage.2016; Accessed 10th August 2017. Reference Source

[9] Agarwal S Perry HB Long LA: Evidence on feasibility and effective use of mHealth strategies by frontline health workers in developing countries: systematic review. *Trop Med Int Health.* 2015;20(8):1003–14. 10.1111/tmi.12525 25881735 PMC4692099

[10] Yousuf Hussein S Wet Swanepoel D Biagio de Jager L: Smartphone hearing screening in mHealth assisted community-based primary care. *J Telemed Telecare.* 2016;22(7):405–12. 10.1177/1357633X15610721 26468215

[11] Bastawrous A Rono HK Livingstone IA: Development and Validation of a Smartphone-Based Visual Acuity Test (Peek Acuity) for Clinical Practice and Community-Based Fieldwork. *JAMA Ophthalmol.* 2015;133(8):930–7. 10.1001/jamaophthalmol.2015.1468 26022921 PMC5321502

[12] Tian M Ajay VS Dunzhu D: A Cluster-Randomized, Controlled Trial of a Simplified Multifaceted Management Program for Individuals at High Cardiovascular Risk (SimCard Trial) in Rural Tibet, China, and Haryana, India. *Circulation.* 2015;132(9):815–24. 10.1161/CIRCULATIONAHA.115.015373 26187183 PMC4558306

[13] Ashley EA Dhorda M Fairhurst RM: Spread of artemisinin resistance in *Plasmodium falciparum* malaria. *N Engl J Med.* 2014;371(5):411–23. 10.1056/NEJMoa1314981 25075834 PMC4143591

[14] Noedl H Se Y Schaecher K: Evidence of artemisinin-resistant malaria in western Cambodia. *N Engl J Med.* 2008;359(24):2619–20. 10.1056/NEJMc0805011 19064625

[15] Dondorp AM Nosten F Yi P: Artemisinin resistance in *Plasmodium falciparum* malaria. *N Engl J Med.* 2009;361(5):455–67. 10.1056/NEJMoa0808859 19641202 PMC3495232

[16] Dunachie S Ngor P White LJ: Pre-implementation survey. *figshare.*Dataset.2018 10.6084/m9.figshare.6326723

[17] Dunachie S Ngor P White LJ: Post-implementation survey. *figshare.*Paper.2018 10.6084/m9.figshare.6327221

[18] Dunachie S Ngor P Chalk J: Code for Smartphone app. *figshare.*Code.2018 10.6084/m9.figshare.6353210

