# Supplementary material for: Smartphones for community health in rural Cambodia: A feasibility study
Source: Wellcome Open Res. 2018 Jun 12;3:69. [Version 1] doi: 10.12688/wellcomeopenres.13751.1 (PMC6069733; doi:10.12688/wellcomeopenres.13751.1)
Supplement: Supplementary file 1 [file wellcomeopenres-3-14942-s0000.tgz › 5a003572-7c83-4e5f-9082-4c14cc358900.docx]

**Smartmal: Smartphones for community health in rural Cambodia**

**Questionnaire for VMR before introduction of smartphones**

5^th^ August 2014

**A. Information about you**

Name ______________________________________

Village _______________________________________

District _______________________________________

1. Man Woman
2. Age ___________
3. Job _______________________
4. Education

- Never attended
- Some primary
- Completed primary
- Some secondary
- Completed secondary
- More than secondary

1. How long have you been a Village Malaria Worker?

- I am a new Village Malaria Worker
- Less than 6 months
- 6 months to 1 year
- 1 – 5 years
- >5 - 10 years
- >10 years

1. Ethnicity ____________________

**B. Information about you and phones**

A mobile phone is a simple phone that can be used to make phone calls and to send text messages

A smartphone is a more complex phone with a larger colour screen and you can use applications (“apps”) to send and receive information, play games etc.

1. Which one of the following describes your experience with mobile phones?

- I have my own mobile phone
- I can use a mobile phone belonging to someone else sometimes
- I have used a mobile phone before but at present I don’t have any access to one
- I have never used a mobile phone

1. Which one of the following describes your experience with smartphones?

- I have my own smartphone
- I can use a smartphone belonging to someone else sometimes
- I have used a smartphone before but at present I don’t have any access to one
- I have never used a smartphone

1. If you have used a smartphone before, please choose all the sentences below which are true:

- I have used a smartphone to make a phonecall
- I have used a smartphone to send or receive text messages
- I have used a smartphone to send or receive emails
- I have used a smartphone to watch videos
- I have used a smartphone to play games
- I have used a smartphone to read the internet
- I have used a smartphone to use apps

1. Have you ever used a mobile phone as part of your role as a village malaria worker?
2. Have you ever used a smartphone as part of your role as a village malaria worker?
3. How do you feel about being given a smartphone for your work as a village malaria worker? Do you feel any of the following about getting a smartphone for your work as a village malaria worker?

- I think it will make my job easier
- I think it will make my job harder
- I will be excited to use a smartphone for my work
- I will be proud to use a smartphone for my work
- I am worried I will find it difficult to use
- I am worried it might get lost or broken
- I am worried someone will steal it from me
- I am worried there will be problems charging it
- Other ____________________

1. Have you ever used a solar power charger?
2. How do you feel about being given a solar power charger for your work as a village malaria worker? Do you feel any of the following about getting a solar power charger for your work as a village malaria worker?

- I think it will make my job easier
- I think it will make my job harder
- I will be excited to use a smartphone for my work
- I will be proud to use a smartphone for my work
- I am worried I will find it difficult to use
- I am worried it might get lost or broken
- I am worried someone will steal it from me
- I am worried there will be problems charging it
- Other ____________________

**C. Information about your role as a village malaria worker**

1. What is your motivation for being a village malaria worker?
   - To help others
   - Contribution to malaria control
   - People respect my work
   - Free services in the HC
   - Financial incentives
   - Non- financial incentives
   - Attain new skills and knowledge
   - Other ____________________
2. What are the main challenges of being a village malaria worker?

- Long distance to patient’s home
- Lack of transportation
- Not enough time
- Too many other tasks
- Lack of incentives
- Poor patient adherence/cooperation
- Misunderstanding about malaria
- Not enough support from HC
- Other ____________________

1. Approximately, how often do you attend Health Centre meetings for village malaria workers?

- Every month
- 6 times a year
- 4 times a year
- 2 times a year
- Yearly
- Never

1. Reason for missing monthly meetings: _____________________________
2. Do you ever have stock-outs for the rapid detection test for malaria?

Yes No

1. Do you ever have stock-outs for the drug treatment for malaria?

Yes No

1. If you experience stock-outs, what do you think the reason is:

_______________________________________

1. In your village, what is your estimate of the proportion of people with fever come to you?

- All
- Most
- Some
- Nobody

1. If people come to you with fever, what do you do if the rapid diagnostic test for malaria is negative?

- Tell them they don’t have Malaria
- Tell them to go home
- Tell them to go to the HC
- Other ______________________________

1. Do people in your village ask you for advice about medical problems apart from fever?

Yes No

1. If yes, what do you say:

- Say that I cannot help
- Tell them to go to the Health Centre
- Tell them to see someone else in the village
- Other

1. How do you feel when you are unable to help?

- I want to help people more but don’t know how
- I want to learn more about healthcare
- I feel frustrated
- I feel people respect me less
- It is not my problem

Date attended: _____________________________
