# Supplementary material for: Smartphones for community health in rural Cambodia: A feasibility study
Source: Wellcome Open Res. 2018 Jun 12;3:69. [Version 1] doi: 10.12688/wellcomeopenres.13751.1 (PMC6069733; doi:10.12688/wellcomeopenres.13751.1)
Supplement: Supplementary file 2 [file wellcomeopenres-3-14942-s0001.tgz › 1e6daaf0-b057-4cb3-be74-76c85b930d0f.docx]

| NO. | QUESTIONS AND FILTERS | CODING CATEGORIES |
| --- | --- | --- |
| Q1 | Do you still have the smartphone that you  were given? | Yes 1  No 2 |
| Q1a | If No, what happened to the smartphone? | Lost 1  Stolen 2  Other 3 __________________________________ |
| Q2 | Have you been able to charge the smartphone using the solar power? | Yes 1  No 2 |
| Q2a | If No, what stopped you? | I tried but it would not charge 1  The solar power was stolen 2  The solar power is broken 3  The solar panel is not in the sunlight 4 |
| Q3 | If everything is working, have you been able  to send the SMS? | Yes 1  No 2 |
| Q3a | If No, what stopped you? | No Malaria cases to report 1  I forgot to send it 2  Need more training 3  Other 4 ______________________________________ |
| Q4 | **For Memot VMWs only**  Do you find using the smartphone easier to use than the old feature phone (Nokia)? | Yes 1  No 2 |
| Q5 | Do you have any comments about using the smartphones? |  |
| Q6 | What do people in your village say about the smartphone? |  |
