# Supplementary material for: Smartphones for community health in rural Cambodia: A feasibility study
Source: Wellcome Open Res. 2018 Jun 12;3:69. [Version 1] doi: 10.12688/wellcomeopenres.13751.1 (PMC6069733; doi:10.12688/wellcomeopenres.13751.1)
Supplement: Peer review history

# Peer review: Referee response for version 1

**DOI:** 10.21956/wellcomeopenres.14942.r33433

**Published:** 2018-07-27

**Authors:** Jannie Hugo

**Affiliations:** 1 Department of Family Medicine, School of Medicine, University of Pretoria, Pretoria, South Africa

Thank you for the opportunity to review the article.

 The article is relevant for the file of community oriented primary care (COPC). The use of smartphones is essential for this work. The study is well designed and clearly reported.

 An issue that is not addressed in the article is how the information on the cell phone is used. An important aspect of the use of mobile ICT is the way in which data is used to inform and adapt practice. It is not good enough for data to be used for reporting purposes only, it should guide practice. Another issue is how the system facilitate clinical support for the community based workers.

 The authors can consider to mention these issues in the article and state how it is addressed or how a future study can address that. 

 Otherwise the article is acceptable for indexing.

I have read this submission. I believe that I have an appropriate level of expertise to confirm that it is of an acceptable scientific standard.

# Peer review: Referee response for version 1

**DOI:** 10.21956/wellcomeopenres.14942.r33291

**Published:** 2018-07-09

**Authors:** Roma Chilengi

**Affiliations:** 1 Centre for Infectious Disease Research in Zambia (CIDRZ), Lusaka, Zambia

Overall, I felt the article was well-written and succinct, identifying a relevant improvement to support the work of an important niche health service provider (the VMW). Data collection for the study involved administration of a pre-study questionnaire, and in person questionnaire to collect qualitative and quantitative data on volunteers’ experiences. Outcome measures were (1) Survival of smart phones and chargers over 12 months post-study commencement (as determined by inspection of the equipment by CNM staff) and (2) acceptability of smart phone use by VMWs (emerging themes from the in-person questionnaires were collated).

 The study made the assertion “ **the current study expands the literature to demonstrate feasibility and acceptability in an impoverished population of Cambodian CHWs, to support malaria control strategies**.” In my view, however, the methods were not sufficient to capture some key feasibility and accessibility information. Furthermore, it appears that in some communities, the role of VMW is becoming obsolete in some areas “ **In the context of their current role in villages with reduced malaria, villagers presenting with fever are frequently not consulting VMWs.” **The authors acknowledge the limitations posed by the small number of VMWs in the pilot study, the short duration of follow-up, and the restricted amount of information collected on follow-up, and also suggest the necessity to enlarge the role of VMWs to include more CHW responsibilities.

Recommendation: the authors might consider demonstrating how readily the VMW role could be expanded to a more general CHW role, which to me would ably make the case for feasibility. Most developing countries are plagued by a range of infectious diseases and finding broader public health use of technology would be more beneficial.

**Assessing feasibility of the proposed implementation**

 The study was conducted in two health areas in communities that had different malaria profiles, with VMWs who had differing levels of prior exposure to smart phones, which makes the results feel more compelling. Literacy is a requirement for being a VMW, which may not be the case for community health workers in other settings.

Recommendatio **n**: the authors might consider discussing the ramifications of deploying the app more generally in the context of the literacy requirement.

**Assessing technological and system capabilities/requirements**

 Implementation required provision of a smart phone, solar-powered smart phone charger and three hours of instruction.

 This amount of training seemed to be adequate and effective, even in the cohort which had little prior exposure to smart phones, which suggests good feasibility in terms of skills transfer/capacity building. The study was carried out over a 12-month period, with the majority phones and chargers being found to be in good condition after that interval, further making the argument for feasibility. The authors acknowledged the short time frame (given the high cost, it would be expected that the lifetime of the phone would need to be significantly higher than one year). However, usage and storage conditions were not specifically addressed, and it was not clear to what extent smart phone durability was tested in real world conditions. If the expectation is that the phone systems are kept in a locked cabinet in the clinic the majority of the time, this may affect feasibility.

Recommendation **:** the authors might consider addressing how and when phones were accessible to the VMWs.

Advantages: The mobile app is simple to use, intuitive and does not require current network access to be operational. The software code open source and available online, making the study intervention accessible and replicable in that respect. The availability of 99% mobile data coverage in the study setting made it feasible for this study, but would perhaps be a concern for other remote health areas interested in trying this model.

Recommendation: the authors might consider discussing the practicalities of implementation in settings with less robust mobile date coverage (however, this may be considered to be outside of the scope of the study)

Concerns

1. The cost of USD175 per VMW seemed prohibitive to scaling. The article reports that “ **the numbers were limited by the cost of the phones”** a challenge which lies at the heart of feasibility.
Recommendation: the authors might consider addressing how this cost would be managed (the use of “supported schemes” is mentioned early in the text but not elaborated upon when referring to the plan for staged introduction of phones).
2. The authors chose to combine the findings of pride and excitement/proud to be using the phone. However, this concealed the fact that when disaggregated, the numbers for each category were actually quite low: My concern was that the study may not have adequately captured concerns relating to the use of the smart phone (given the closed nature of the questionnaire), and that problems might therefore be replicated. I would be interested to know why the 2 VMWs used paper records rather than the phones, and whether, if the other 5 VMWs had no cases to report, is introducing an expensive cellphone really feasible? I would further be interested in gaining more specific information about the uptake and use of the technology in the village with high numbers of reporting, and to what extent paper reports were generated in that setting, which is not elaborated in the text.
3. I felt that the study did not capture how the use of phones would/did make the work easier: I assume it would be related to data reporting, but this is not specified in the article. While the study was able to record that VMWs *felt* the smart phones would allow faster communication, it is not specified if that was actually the case. The methods used did not capture this question, and it is not clear if that was assessed or determined in any way.
4. The authors make the assertion **: “we have demonstrated smartphone technology to be a robust platform for delivery of these services in the local language, enabling use of voice calls, sms, photography, videos, audio and gps tracking**.” Recommendation: the authors might consider disaggregating the data from the group which reported a large number of malaria cases using the smartphones to more strongly support this assertion.

I have read this submission. I believe that I have an appropriate level of expertise to confirm that it is of an acceptable scientific standard, however I have significant reservations, as outlined above.

# Peer review: Referee response for version 1

**DOI:** 10.21956/wellcomeopenres.14942.r33292

**Published:** 2018-07-05

**Authors:** Gerry F. Killeen

**Affiliations:** 1 Environmental Health and Ecological Sciences Thematic group, Ifakara Health Institute, Dar es Salaam, Tanzania; 2 Department of Vector Biology, Liverpool School of Tropical Medicine, Liverpool, UK

Overall, a very well written and useful paper delivering useful and practically-relevant evidence.

**Major comment**

1. The need to shift from VMWs to CHWs is clearly spelled out in the final paragraph of the discussion, but needs to be more clearly and explicitly stated in the abstract.

**Minor comments**

1. The following sentence from the second paragraph of the results section is vague: “100% were literate (a requirement for being a VMW) with the highest level of education completed being primary school level only for 81%”. Does that mean that 81% had not completed primary school, yet were literate? Or that 81% had primary education and the remainder has secondary or above? I’m guessing the word “only may have been misplaced in this sentence.
2. Third paragraph of the results section: It is a little unclear whether the smart phone with the damaged USB port is the one counted as not working well. If so, I think it would be worthwhile to make it clear that all 27 were still in place.
3. The first sentence of the second paragraph of the discussion is too long. Please split in two and make it read easier.

I have read this submission. I believe that I have an appropriate level of expertise to confirm that it is of an acceptable scientific standard, however I have significant reservations, as outlined above.
